# *Pelecotoidessinicus*, a new species of ripiphorid beetle from east China (Ripiphoridae, Ptilophorinae)

**DOI:** 10.3897/BDJ.13.e158408

**Published:** 2025-07-15

**Authors:** Lan-Xing Jiang, Yuchen Zhao, Xiu-Min Li, Zhao Pan

**Affiliations:** 1 Key Laboratory of Zoological Systematics and Application of Hebei Province, School of Life Sciences, Institute of Life Science and Green Development, Hebei University, Baoding, China Key Laboratory of Zoological Systematics and Application of Hebei Province, School of Life Sciences, Institute of Life Science and Green Development, Hebei University Baoding China

**Keywords:** wedge-shaped beetles, Ptilophorinae, new species, China.

## Abstract

**Background:**

The ripiphorid genus *Pelecotoides* Laporte, 1833 is distributed primarily throughout the Southern Hemisphere, with approximately 72 species. Although exhibiting robust species richness, only one *Pelecotoides* species, *P.tokejii* (Nomura & Nakane, 1959), has been recorded from Asia.

**New information:**

Herein, *Pelecotoidessinicus* Jiang & Pan **sp. nov.** is described and illustrated from Zhejiang and Fujian, China, representing the second Asian species of *Pelecotoides*. This new species can be easily distinguished from *P.tokejii* by its sexually dimorphic antennae.

## Introduction

*Pelecotoides* Laporte, 1833, previously known widely as *Trigonodera* Dejean, 1834 (see Batelka et al. (2025)), is the most speciose genus within the subfamily Ptilophorinae Gerstaecker, 1855. The genus includes approximately 72 species and occurs largely throughout South America, Australia and Africa (Falin 2002, Lawrence et al. 2010, Batelka and Chaboo 2015). However, it remains inadequately studied and is in need of complete revision. As defined in previous literature (Gerstaecker 1855, Batelka 2009, Batelka 2012), *Pelecotoides* is distinguished from other genera of Ptilophorinae by the combination of the following characters: male antennae short pectinate or serrate, basal antennomeres cylindrical; maxillary palpomere III shortest, labial palpomeres II and III subequal in length, ligula subcordate; eyes slightly emarginate; tibial spur formula 2-2-2, pretarsal claws with pectinate teeth along ventral face. The natural history of this genus is poorly known, except for observations that adults visit flowers during the day, exhibit positive phototaxis at night and can be collected by Malaise and light traps or by beating trees (Falin 2002, Hsiao and Huang 2017).

**Figure 1.**
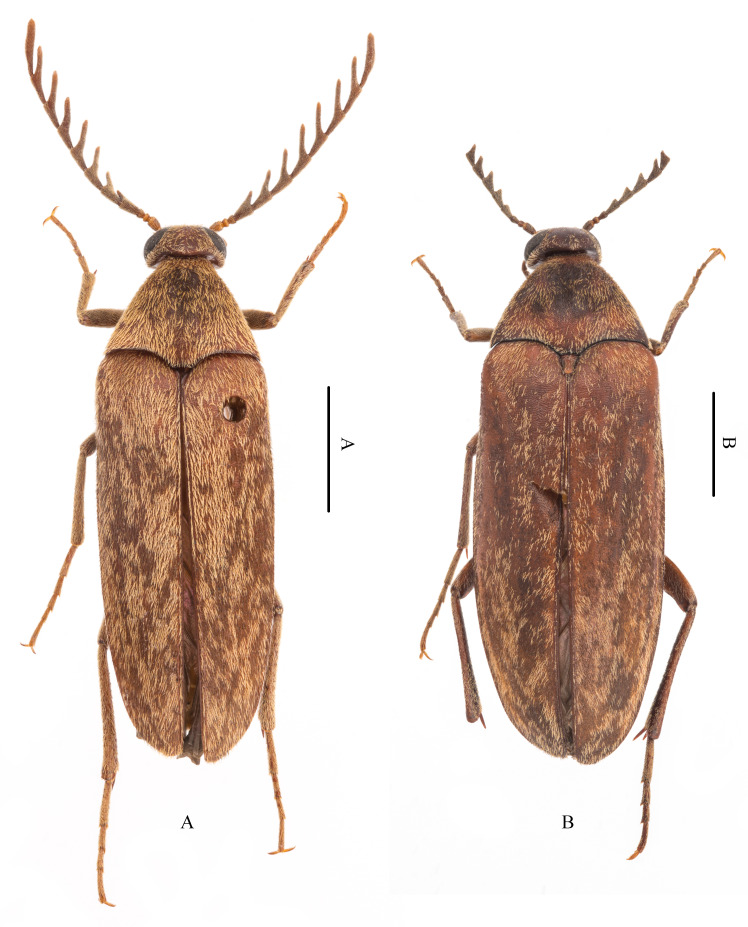
Habitus of *Pelecotoidessinicus* Jiang & Pan, sp. nov., paratypes, dorsal view. **A** Male; **B** Female. Scale bar: 2 mm.

Although exhibiting robust species richness, *Pelecotoides* is known from Asia by only one species, *Pelecotoidestokejii* (Nomura & Nakane, 1959). Asian records include Japan and Taiwan, China (Nomura and Nakane 1959, Hsiao and Huang 2017). While examining Chinese ripiphorids, we discovered a new species, *Pelecotoidessinicus* Jiang & Pan, **sp. nov.**, from Zhejiang and Fujian Provinces (Figs. 1A–1B). The new species is described and illustrated below.

## Materials and methods

The type material is deposited in the Museum of Hebei University, Baoding, China (MHBU) and the Collection of Forest Entomology Laboratory, Beijing Forestry University, Beijing, China (FELC), respectively. Images were produced using a Canon EOS 5D Mark III (Canon Inc., Tokyo, Japan) connected to a Laowa EF 100 mm F2.8 CA-Dreamer Macro 2× or Laowa EF 25 mm F2.8 Ultra Macro 2.5–5× (Anhui Changgeng Optics Technology Co., Ltd., Hefei, China).

The terms for male genitalia are based on those presented in Lawrence and Ślipiński (2013). Other morphological terms in the descriptions generally adhere to the previous literature (Nomura and Nakane 1959, Falin 2002).

## Taxon treatments

### Pelecotoides sinicus

Jiang & Pan sp. nov.

376DF1BF-B3B0-5905-A5B7-81BACD70719C

#### Materials

- **Type status:** Holotype. **Occurrence:** recordedBy: Cai-Xia Yuan; Di Li; sex: male; lifeStage: adult; establishmentMeans: wild; occurrenceID: E9C60014-7469-594A-BAAB-C07355EA36B4; **Taxon:** kingdom: Animalia; phylum: Arthropoda; class: Insecta; order: Coleoptera; family: Ripiphoridae; genus: Pelecotoides ; specificEpithet: *sinicus*; taxonRank: species; verbatimTaxonRank: sp.; scientificNameAuthorship: Jiang & Pan; nomenclaturalCode: ICZN; taxonomicStatus: accepted; nomenclaturalStatus: sp. nov; **Location:** continent: Asia; country: China; countryCode: China/CN; stateProvince: Zhejiang; county: Lin’an; locality: Tianmu Mountain Administration Bureau; verbatimElevation: 320 m; verbatimLatitude: 30d 18' 52.98" N; verbatimLongitude: 119d 26' 29.50" E; verbatimCoordinateSystem: degrees minutes seconds; verbatimSRS: GCJ02; **Identification:** identifiedBy: Pan Z; Jiang L-X; dateIdentified: 2024; **Event:** year: 2014; month: 7; day: 20; **Record Level:** type: PhysicalObject; language: en; rightsHolder: Museum of Hebei University; institutionCode: MHBU
- **Type status:** Paratype. **Occurrence:** recordedBy: Cai-Xia Yuan; Di Li; individualCount: 2; sex: 1 male, 1 female; lifeStage: adult; establishmentMeans: wild; occurrenceID: 94513128-F5A1-54DD-9A5F-09690CAEF37D; **Taxon:** kingdom: Animalia; phylum: Arthropoda; class: Insecta; order: Coleoptera; family: Ripiphoridae; genus: Pelecotoides ; specificEpithet: *sinicus*; taxonRank: species; verbatimTaxonRank: sp.; scientificNameAuthorship: Jiang & Pan; nomenclaturalCode: ICZN; taxonomicStatus: accepted; nomenclaturalStatus: sp. nov.; **Location:** continent: Asia; country: China; countryCode: China/CN; stateProvince: Zhejiang; county: Lin'an; locality: Tianmu Mountain Administration Bureau; verbatimElevation: 320 m; verbatimLatitude: 30d 18' 52.98" N; verbatimLongitude: 119d 26' 29.50" E; verbatimCoordinateSystem: degrees minutes seconds; verbatimSRS: GCJ02; **Identification:** identifiedBy: Pan Z; Jiang L-X; dateIdentified: 2024; **Event:** year: 2014; month: 7; day: 20; **Record Level:** type: PhysicalObject; language: en; rightsHolder: Museum of Hebei University; institutionCode: MHBU
- **Type status:** Paratype. **Occurrence:** recordedBy: Cai-Xia Yuan; Di Li; individualCount: 3; sex: 1 male, 2 females; lifeStage: adult; establishmentMeans: wild; occurrenceID: AC935F3F-2C3D-5BE7-8194-B87F4E15F637; **Taxon:** kingdom: Animalia; phylum: Arthropoda; class: Insecta; order: Coleoptera; family: Ripiphoridae; genus: Pelecotoides ; specificEpithet: *sinicus*; taxonRank: species; verbatimTaxonRank: sp.; scientificNameAuthorship: Jiang & Pan; nomenclaturalCode: ICZN; taxonomicStatus: accepted; nomenclaturalStatus: sp. nov.; **Location:** continent: Asia; country: China; countryCode: China/CN; stateProvince: Zhejiang; county: Lin’an; locality: Tianmu Mountain Administration Bureau; verbatimElevation: 320 m; verbatimLatitude: 30d 18' 52.98" N; verbatimLongitude: 119d 26' 29.50" E; verbatimCoordinateSystem: degrees minutes seconds; verbatimSRS: GCJ02; **Identification:** identifiedBy: Pan Z; Jiang L-X; dateIdentified: 2024; **Event:** year: 2014; month: 7; day: 18; **Record Level:** type: PhysicalObject; language: en; rightsHolder: Museum of Hebei University; institutionCode: MHBU
- **Type status:** Paratype. **Occurrence:** individualCount: 1; sex: female; lifeStage: adult; establishmentMeans: wild; occurrenceID: 215C3DD9-AB5C-565A-BEC2-10FD1AF0EACE; **Taxon:** kingdom: Animalia; phylum: Arthropoda; class: Insecta; order: Coleoptera; family: Ripiphoridae; genus: Pelecotoides ; specificEpithet: *sinicus*; taxonRank: species; verbatimTaxonRank: sp.; scientificNameAuthorship: Jiang & Pan; nomenclaturalCode: ICZN; taxonomicStatus: accepted; nomenclaturalStatus: sp. nov.; **Location:** continent: Asia; country: China; countryCode: China/CN; stateProvince: Zhejiang; county: Lin’an; locality: Tianmu Mountain; **Identification:** identifiedBy: Pan Z; Jiang L-X; **Record Level:** type: PhysicalObject; language: en; rightsHolder: Collection of Forest Entomology Laboratory; institutionCode: FELC
- **Type status:** Paratype. **Occurrence:** recordedBy: Wen-Xuan Bi; individualCount: 1; sex: female; lifeStage: adult; establishmentMeans: wild; occurrenceID: A946434B-3DB7-5B50-BD66-8DC6EF54FDC6; **Taxon:** kingdom: Animalia; phylum: Arthropoda; class: Insecta; order: Coleoptera; family: Ripiphoridae; genus: Pelecotoides ; specificEpithet: *sinicus*; taxonRank: species; verbatimTaxonRank: sp.; scientificNameAuthorship: Jiang & Pan; nomenclaturalCode: ICZN; taxonomicStatus: accepted; nomenclaturalStatus: sp. nov.; **Location:** continent: Asia; country: China; countryCode: China/CN; stateProvince: Zhejiang; county: Lin’an; locality: West Tianmu Mountain; verbatimElevation: 300-400 m; **Identification:** identifiedBy: Pan Z; Jiang L-X; **Event:** year: 2006; month: 7; day: 27-29; **Record Level:** type: PhysicalObject; language: en; rightsHolder: Collection of Forest Entomology Laboratory; institutionCode: FELC
- **Type status:** Paratype. **Occurrence:** recordedBy: Hong-Liang Shi; individualCount: 3; sex: female; establishmentMeans: wild; occurrenceID: 63B41E0E-30B2-5B37-9E5E-94CFB275927E; **Taxon:** kingdom: Animalia; phylum: Arthropoda; class: Insecta; order: Coleoptera; family: Ripiphoridae; genus: Pelecotoides ; specificEpithet: *sinicus*; taxonRank: species; verbatimTaxonRank: sp.; scientificNameAuthorship: Jiang & Pan; nomenclaturalCode: ICZN; taxonomicStatus: accepted; nomenclaturalStatus: sp. nov.; **Location:** continent: Asia; country: China; countryCode: China/CN; stateProvince: Zhejiang; county: Lin’an; locality: West Tianmu Mountain; verbatimElevation: 300-400 m; verbatimLatitude: 30d 19′ N; verbatimLongitude: 119d 27′ E; verbatimCoordinateSystem: degrees decimal minutes; verbatimSRS: GCJ02; **Identification:** identifiedBy: Pan Z; Jiang L-X; **Event:** samplingProtocol: light trap; year: 2006; month: 8; day: 18; **Record Level:** type: PhysicalObject; language: en; rightsHolder: Collection of Forest Entomology Laboratory; institutionCode: FELC
- **Type status:** Paratype. **Occurrence:** recordedBy: Hong-Liang Shi; individualCount: 1; sex: female; lifeStage: adult; establishmentMeans: wild; occurrenceID: 4A50C92D-5BA9-50AE-A14B-88BD17F14AFE; **Taxon:** kingdom: Animalia; phylum: Arthropoda; class: Insecta; order: Coleoptera; family: Ripiphoridae; genus: Pelecotoides ; specificEpithet: *sinicus*; taxonRank: species; verbatimTaxonRank: sp.; scientificNameAuthorship: Jiang & Pan; nomenclaturalCode: ICZN; taxonomicStatus: accepted; nomenclaturalStatus: sp. nov.; **Location:** continent: Asia; country: China; countryCode: China/CN; stateProvince: Zhejiang; county: Lin’an; locality: West Tianmu Mountain; verbatimElevation: 300-400 m; verbatimLatitude: 30d 19′ N; verbatimLongitude: 119d 27′ E; verbatimCoordinateSystem: degrees decimal minutes; verbatimSRS: GCJ02; **Identification:** identifiedBy: Pan Z; Jiang L-X; **Event:** samplingProtocol: light trap; year: 2006; month: 8; day: 20; **Record Level:** type: PhysicalObject; language: en; rightsHolder: Collection of Forest Entomology Laboratory; institutionCode: FELC
- **Type status:** Paratype. **Occurrence:** recordedBy: Jia-Chen Zhu; Xiao-Fei Li; Ze-Kai Li; individualID: R1B1; R1B2; individualCount: 2; sex: male; lifeStage: adult; establishmentMeans: wild; occurrenceID: 07F941AA-4388-5DC0-A7D8-8151541E3D71; **Taxon:** kingdom: Animalia; phylum: Arthropoda; class: Insecta; order: Coleoptera; family: Ripiphoridae; genus: Pelecotoides ; specificEpithet: *sinicus*; taxonRank: species; verbatimTaxonRank: sp.; scientificNameAuthorship: Jiang & Pan; nomenclaturalCode: ICZN; taxonomicStatus: sp. nov.; **Location:** continent: Asia; country: China; countryCode: China/CN; stateProvince: Zhejiang; county: Lin'an; locality: Tianmu Mountain, Qiangwei Garden; verbatimElevation: 300 m; **Identification:** identifiedBy: Pan Z; Jiang L-X; **Event:** samplingProtocol: light trap; year: 2019; month: 7; day: 25; **Record Level:** type: PhysicalObject; language: en; rightsHolder: Museum of Hebei University; institutionCode: MHBU
- **Type status:** Paratype. **Occurrence:** recordedBy: Jia-Chen Zhu; Xiao-Fei Li; Ze-Kai Li; individualCount: 1; sex: male; lifeStage: adult; establishmentMeans: wild; occurrenceID: A2525EDE-7722-57BE-9BB8-DC485D0B2300; **Taxon:** kingdom: Animalia; phylum: Arthropoda; class: Insecta; order: Coleoptera; family: Ripiphoridae; genus: Pelecotoides ; specificEpithet: *sinicus*; taxonRank: species; verbatimTaxonRank: sp.; scientificNameAuthorship: Jiang & Pan; nomenclaturalCode: ICZN; taxonomicStatus: accepted; nomenclaturalStatus: sp. nov.; **Location:** continent: Asia; country: China; countryCode: China/CN; stateProvince: Zhejiang; county: Lin’an; locality: Tianmu Mountain, Qiangwei Garden; verbatimElevation: 300 m; **Identification:** identifiedBy: Pan Z; Jiang L-X; **Event:** samplingProtocol: light trap; year: 2019; month: 7; day: 26; **Record Level:** type: PhysicalObject; language: en; rightsHolder: Museum of Hebei University; institutionCode: MHBU
- **Type status:** Paratype. **Occurrence:** recordedBy: Sai-Hong Dong; Shan-Shan Liu; individualCount: 1; sex: female; occurrenceID: BFB4B97D-8E14-5807-A4B8-B8EDBA2FA006; **Taxon:** kingdom: Animalia; phylum: Arthropoda; class: Insecta; order: Coleoptera; family: Ripiphoridae; genus: Pelecotoides ; specificEpithet: *sinicus*; taxonRank: species; verbatimTaxonRank: sp.; scientificNameAuthorship: Jiang & Pan; nomenclaturalCode: ICZN; taxonomicStatus: accepted; nomenclaturalStatus: sp. nov.; **Location:** continent: Asia; country: China; countryCode: China/CN; stateProvince: Zhejiang; county: Lin'an; locality: Tianmu Mountain, Chanyuan Temple; verbatimElevation: 397 m; verbatimLatitude: 30d 19′ 27.07″ N; verbatimLongitude: 119d 26′ 29.52″ E; verbatimCoordinateSystem: degrees minutes seconds; verbatimSRS: GCJ02; **Identification:** identifiedBy: Pan Z; Jiang L-X; **Event:** year: 2014; month: 7; day: 19; **Record Level:** type: PhysicalObject; language: en; rightsHolder: Museum of Hebei University; institutionCode: MHBU
- **Type status:** Paratype. **Occurrence:** recordedBy: Hong-Liang Shi; Gan-Yan Yang; Zhi-Duo Shi; individualCount: 1; sex: female; lifeStage: adult; establishmentMeans: wild; occurrenceID: F34544F8-FC40-5F50-8759-B6444ACDD70C; **Taxon:** kingdom: Animalia; phylum: Arthropoda; class: Insecta; order: Coleopatera; family: Ripiphoridae; genus: Pelecotoides ; specificEpithet: *sinicus*; taxonRank: species; verbatimTaxonRank: sp.; scientificNameAuthorship: Jiang & Pan; nomenclaturalCode: ICZN; taxonomicStatus: accepted; nomenclaturalStatus: sp. nov.; **Location:** continent: Asia; countryCode: China/CN; stateProvince: Fujian; county: Wuping; municipality: Yanqian Township; locality: Butterfly Valley; verbatimElevation: 400 m; verbatimLatitude: 26.3133d N; verbatimLongitude: 117.5003d E; verbatimCoordinateSystem: decimal degrees; verbatimSRS: GCJ02; **Identification:** identifiedBy: Pan Z; Jiang L-X; **Event:** year: 2024; month: 10; day: 3; **Record Level:** type: PhysicalObject; language: en; rightsHolder: Museum of Hebei University; institutionCode: MHBU

#### Description

Male (Fig. 1A): Body length: 5.7–8.5 mm, humeral width: 2.1–2.8 mm (n = 6); reddish-brown, except frons, occiput, centre of pronotum, mesanepisternum and tibial spurs dark, approximately apical 1/4 of mandibles black, antennomeres I–III lighter than remaining antennomeres, abdominal ventrites usually yellowish-brown; body densely covered with yellow setae partially lacking in a few individuals due to poor preservation.

**Figure 2.**
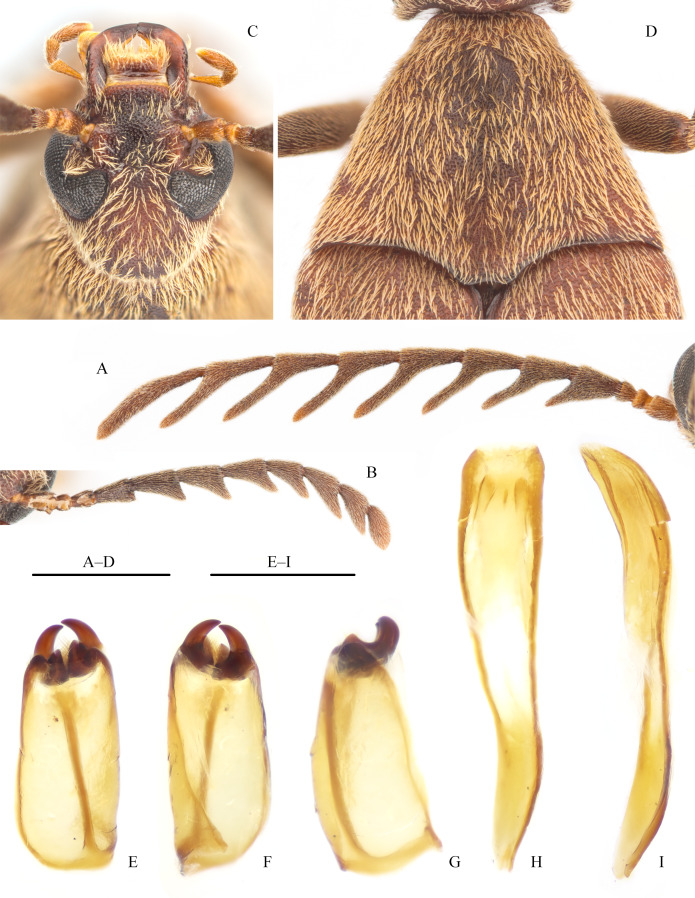
Characters of *Pelecotoidessinicus* Jiang & Pan, sp. nov., paratypes. **A, B** Antennae of male (A) and female (B); **C** Head; **D** Pronotum; **E–G** Tegmen; dorsal view (E), ventral view (F) and lateral view (G); **H–I** Median lobe; dorsal view (H) and lateral view (I). Scale bars: 1 mm (A–D); 0.5 mm (E–I).

Head (Fig. 2C) longer (1.83 mm) than wide (1.38 mm), with small and dense punctures; frons slightly depressed; frontoclypeal suture obsolete; anterior clypeal margin straight; labrum transverse, anterior margin slightly emarginate mesally; mandibles long, sickle-shaped; maxillary palpomere II approximately as long as, but narrower than IV. Anterior margins of compound eyes distinctly emarginate to accommodate antennal fossae. Antennae (Fig. 2A) approximately 1.85× pronotal length, with 11 antennomeres; antennomeres I–III subcylindrical, antennomere I widened apically, antennomeres II–III short, antennomere II shortest; antennomeres IV–X short pectinate; length ratio between each antennomere and shorest one (II) as follows: I: 1.9; III: 1.1; IV: 3.0; V: 3.6; VI: 4.0; VII: 4.0; VIII: 4.0; IX: 4.0; X: 4.7; XI: 8.5; length ratio between each ramus of antennomeres IV–X and antennomere II as follows: IV: 3.2; V: 4.3; VI: 5.0; VII: 5.3; VIII: 5.4; IX: 5.4; X: 5.0.

Pronotum (Fig. 2D) approximately as long as head, indistinctly trilobed, posterior angles slightly protruding, median lobe distinctly protruding, with posterior margin mesally emarginate; disc convex at centre, with small, dense punctures; lateral carinae distinct and complete. Scutellar shield ovatel. Elytra long, complete, gradually narrowed posterially, rounded apically, sparsely setose basally. Femora widened; tibiae straight, tibial spur formula 2-2-2, each inner spur longer than outer spur, especially those of mesotibia; pretarsal claws pectinate, each with 6-8 teeth along ventral face.

Abdomen with five, densely punctate ventrites. Posterior margin of ventrite V emarginate mesally. Aedeagus sclerotised (Fig. 2E–I); phallobase asymmetrical, with a brown longitudinal oblique stripe and few apical setae (Fig. 2E–G); parameres highly sclerotised, slightly curved apically, hook-shaped (Fig. 2E-G); median lobe asymmetrical, approximately twice as long as tegmen, gradually narrowed apically, apex pointed (Fig. 2H and I).

Female (Fig. 1B). Similar to male, but body larger, length: 9.2–10.6 mm; humeral width 3.8–4.5 mm (n = 11). Abdominal ventrites dark brown. Antennae (Fig. 2B) shorter, approximately 1.3× pronotal length, antennomeres IV–X serrate; ratio between length (width) of each antennomere and length of antennomere II as follows: I: 2.3 (1.2); II: 1.0 (1.0); III: 1.7 (1.0); IV: 2.5 (1.5); V: 3.5 (2.1); VI: 3.2 (2.3); VII: 3.2 (2.3); VIII: 2.7 (2.0); IX: 2.8 (2.3); X: 2.6 (2.0); XI: 3.1 (1.2). Tibial spurs more robust than those of male. Posterior margin of ventrite V straight.

#### Diagnosis

The new species can be easily distinguished from the other Asian *Pelecotoides* species, *P.tokejii*, by the following characters: body (length 5.7–8.5 mm in male, n = 6; 9.2–10.6 mm in female, n = 11) usually smaller than that of *P.tokejii* (length 8.0–15.0 mm in both sexes, Nomura and Nakane (1959), Hsiao and Huang (2017); based on our collections, male length 10.9 mm and female length 12.5 mm, n = 1, respectively); antennae of *P.sinicus* Jiang & Pan, sp. nov. pectinate in male, but serrate in female (Figs. 2A–2B), those of *P.tokejii* serrate in both sexes [figs. 2.1–2.2 in Nomura and Nakane (1959); ratio between the length (width) of each antennomere and length of antennomere II as follows: male: I: 1.5 (0.8); II: 1.0 (0.8); III: 1.3 (1.0); IV: 1.1 (0.8); V: 1.2 (0.9); VI: 1.9 (1.1); VII: 1.8 (1.1); VIII: 1.7 (1.1); IX: 1.6 (1.0); X: 1.5 (1.0); XI: 1.8 (1.1); female: I: 2.3 (1.1); II: 1.0 (0.9); III: 1.5 (0.9); IV: 1.0 (0.8); V: 1.1 (1.0); VI: 1.9 (1.3); VII: 1.8 (1.3); VIII: 1.8 (1.3); IX: 1.7 (1.2); X: 1.5 (1.1); XI: 2.3 (1.1)]; posterior margin of basal pronotal median lobe distinctly emarginate in *P.sinicus* Jiang & Pan, sp. nov. (Fig. 2D), but almost straight in *P.tokejii*.

#### Etymology

The specific name comes from the Latin adjective “*sinicus*” for “Chinese”, in reference to the type locality east China.

#### Distribution

China: Zhejiang, Fujian.

#### Taxon discussion

A comparison of the new species with *P.tokejii* reveals distinct sexual dimorphism, manifesting in the distinct shapes of the body and antenna. The male body is slightly smaller and more slender than the female body. A supplementary note must be made concerning the ratio between the body length and the widest width of the elytra, which is approximately 3.0 mm in males and 2.8 mm in females. In addition, as previously described, the antennae of males are pectinate, while those of females are serrate.

## Supplementary Material

XML Treatment for Pelecotoides sinicus
